# A Simple and Rapid Method for the Selection of Suitable Donors for Transfusion by the Determination of Blood Groups

**Published:** 1918-04

**Authors:** 


***A Simple and Rapid Method for the Selection of Suitable Donors for Transfusion by the Determination of Blood Groups.*** By ROGER I. LEE, Major M. R. C. Army Base Hospital N° 5, B. E. F. Abs. from the *Brit. Med. Jour.*, Nov. 24, 1917.

The most important consideration is not whether donor and recipient belong to the same blood group, but whether the serum of the recipient agglutinates or hemolyzes the red corpuscles of the donor. In the ordinary transfusion an amount equal to only about one-fifth to one-twelfth of the total blood volume of the recipient is added from the donor. The donor's serum is therefore diluted and rendered harmless; furthermore, the patient's cells are protected by his own serum. Any transfusion is consequently permissible provided the patient's serum does not agglutinate and hemolyze the donor's red corpuscles, although the donor's serum may agglutinate and hemolyze the patient's red corpuscles.

| *Serum of Group.* | I | II | III | IV |
|---|---|---|---|---|
| Cells of Group I . . . . . | — | + | + | + |
| " " " II . . . . | o | — | + | + |
| " " " III . . . . | o | + | — | + |
| " " " IV . . . . | o | o | o | — |

Reference to the chart shows that Group I may receive blood from any donor, because its serum will neither hemolyze nor agglutinate the red cells of any group. On the other hand, the cells of Group I are agglutinated and hemolyzed by the serum of any other group but its own (fortunately a small one). Group I is therefore the universal recipient. The blood of Group IV may be transfused with safety into any patient; but, if the patient belongs to Group IV, he can receive blood only from his own group.

Since the sera of persons within the same group vary greatly in agglutinating and hemolytic power, a cross-transfusion which may result in death in one instance may produce only mild symptoms in another.

Though in cases of dire necessity transfusion without tests is permissible, the following procedure may be regarded as the minimum; About 1 cc. of blood is collected from patient's ear or finger and allowed to clot. One drop of the serum obtained is placed upon a slide and mixed with a drop of a suspension of blood of the donor taken into 1.3 per cent citrate solution. (A few drops of blood are taken into ten times the amount of 1.5 citrate solution and shaken. The blood should be dropped *directly* into the citrate and should not be partially coagulated). This test will appear in a few moments and, if positive, marked agglutination will be evident under the microscope. If negative, it is wise to raise the cover glass, make sure that serum and cells are well mixed, and examine again. The only possible source of confusion is the appearance of *rouleaux* of the red corpuscles, indicating too thick an emulsion. If the test be negative, transfusion is safe.

Experience has caused the author to adopt another procedure. He keeps a supply of sera of Groups II, III and IV constantly on hand. If it is known that any person is in either Group II or Group III, all the other groups can be established; it is only necessary to collect the blood of the person to be tested in citrate solution and the test may be performed. If the serum of unknown blood is tested against the known cells, it is necessary to await the development of serum after clotting. Should a known blood be unavailable, a series of bloods may be tested against each other.

*Routine Method.* — With the sera of Groups II, III and IV a number of donors are tested, and, if possible, the patient's blood is also tested in order to use up the donors of groups other than IV. In case of emergency, however, a donor from Group IV is taken and transfusion immediately performed.

Whenever possible preliminary blood tests should be made before transfusion; in their absence fatalities or severe reactions may occur. The test of giving a few cc. of blood and awaiting symptoms of agglutination and hemolysis is unnecessary; it delays transfusion and permits those changes in the blood to occur which eventually lead to coagulation and which so alter the protein molecules of the blood that the blood itself may have very toxic qualities.

Under the conditions set forth above no disastrous effects from transfusion have been observed and no evidence of hemolysis has ever been found.

